# The Effect of Heterozygous Mutation of Adenylate Kinase 2 Gene on Neutrophil Differentiation

**DOI:** 10.3390/ijms232416089

**Published:** 2022-12-17

**Authors:** Taigo Horiguchi, Ayako Tanimura, Keiko Miyoshi, Hiroko Hagita, Hisanori Minami, Takafumi Noma

**Affiliations:** 1 Department of Oral Bioscience, Tokushima University Graduate School of Biomedical Sciences, Tokushima 770-8504, Japan; 2 Division of Food & Health Environmental Sciences, Department of Environmental & Symbiotic Sciences, Faculty of Environmental & Symbiotic Sciences, Prefectural University of Kumamoto, 3-1-100, Tsukide, Higashi-ku, Kumamoto 862-8502, Japan; 3 Division of Applied Nutrition, Faculty of Nutrition, Kobe Gakuin University, 518 Arise, Ikawadani-cho, Nishi-ku, Kobe 651-2180, Japan; 4 Department of Nutrition and Health Promotion, Faculty of Human Life Studies, Hiroshima Jogakuin University, 4-13-1 Ushita-Higashi, Higashi-ku, Hiroshima 732-0063, Japan

**Keywords:** adenylate kinase 2, mitochondria, neutrophil differentiation, reticular dysgenesis, CRISPR/Cas9, fructose

## Abstract

Mitochondrial ATP production plays an important role in most cellular activities, including growth and differentiation. Previously we reported that Adenylate kinase 2 (AK2) is the main ADP supplier in the mitochondrial intermembrane space in hematopoietic cells, especially in the bone marrow. AK2 is crucial for the production of neutrophils and T cells, and its deficiency causes reticular dysgenesis. However, the relationship between ADP supply by AK2 and neutrophil differentiation remains unclear. In this study, we used CRISPR/Cas9 technology to establish two heterozygous AK2 knock-out HL-60 clones as models for reticular dysgenesis. Their AK2 activities were about half that in the wild-type (WT). Furthermore, neutrophil differentiation was impaired in one of the clones. In silico analysis predicted that the obtained mutations might cause a structural change in AK2. Time course microarray analysis of the WT and mutants revealed that similar gene clusters responded to all-trans retinoic acid treatment, but their expression was lower in the mutants than in WT. Application of fructose partially restored neutrophil differentiation in the heterozygous knock-out HL-60 clone after all-trans retinoic acid treatment. Collectively, our study suggests that the mutation of N-terminal region in AK2 might play a role in AK2-dependent neutrophil differentiation and fructose could be used to treat AK2 deficiency.

## 1. Introduction

In our previous study, we set up an in vitro differentiation system using HL-60 cells, derived from a human promyelocytic leukemia patient, to establish two important findings: (i) endoplasmic reticulum stress gets attenuated when neutrophil differentiation is induced, and (ii) ATP supply from mitochondria is critical for neutrophil differentiation [1,2]. For the release of endoplasmic reticulum stress, mitochondrial ATP was required to activate inositol-requiring enzyme-1α, one of the components of the unfolded protein response [2].

ATP production in the mitochondria is regulated in two main ways: (i) by controlling the supply of the substrate, ADP, to adenine nucleotide transporters, which translocate ADP from the intermembrane space into the mitochondrial matrix, (ii) through the activity of the electron transport chain. ADP is supplied to the mitochondrial intermembrane by the action of four types of enzymes: mitochondrial creatine kinases (CKMT1 and CKMT2), nucleoside diphosphate kinase isoform D (NDPK-D), and adenylate kinase 2 (AK2). Among them, AK2 is the main enzyme in the bone marrow [1]. Given that AK2 deficiency causes neutrophil and T cell deficiency, resulting in severe combined immunodeficiency (reticular dysgenesis) [3,4], we focused on the role of AK2 in neutrophil differentiation.

The aim of this study was to investigate the molecular basis for AK2-dependent neutrophil differentiation. Since AK2 knock-out has been reported to be lethal to mouse embryos [5,6], we focused on optimizing an in vitro hematopoietic differentiation assay using the human cell line HL-60. These promyelocytic leukemia cells can differentiate into mature neutrophils upon all-trans retinoic acid (ATRA) treatment, and into macrophages upon phorbol 12-myristate 13-acetate treatment. We used clustered regularly interspaced short palindromic repeats/Cas9 (CRISPR/Cas9) gene-editing to knock-out AK2 in HL-60 cells, and elucidated the effect and detailed mechanisms of AK2 deficiency in this system. We obtained two types of AK2 mutants and discovered a novel structure–function relationship between AK2 mutation and its disordered structure, which is important for regulating neutrophil differentiation. Furthermore, to develop a novel treatment for AK2-deficient patients, we tried rescuing the impaired neutrophil differentiation arising due to the abnormal energy metabolism caused by AK2 disruption. We focused on fructose because it can be metabolized via glycolysis to produce metabolites that enhance ATP production.

Taken together, herein, we have reported the characteristics of AK2-deficient cells by disrupting the AK2 gene.

## 2. Results

### 2.1. Preparation of CRISPR/Cas9-Mediated AK2 Heterozygous Mutants

**Figure 1:**
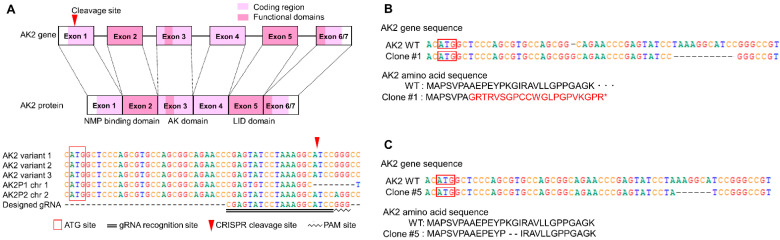
The scheme of adenylate kinase 2 (AK2) gene sequences in clones generated by clustered regularly interspaced short palindromic repeats/Cas9 (CRISPR/Cas9) (**A**) The schematic diagram of the CRISPR/Cas9-targeted site on AK2.AK2 has six or seven exons. The pale pink area indicates the coding region and the dark pink areas indicate functional domains of the AK2 protein. The red arrowhead indicates the Cas9/guide RNA (gRNA)-targeted cleavage site. The bottom shows a sequence alignment of the AK2 gene from AK2 variants 1, 2, 3, and AK2 pseudogene 1 (AK2P1), 2 (AK2P2), and the designed gRNA. The double underline indicates the designed gRNA and the wavy line indicates the protospacer adjacent motif (PAM) site. Chr, chromosome. (**B**) DNA and amino acid mutation sites in AK2 in clone #1 generated from the first trial. Clone #1 was obtained after the first trial of CRISPR/Cas9 treatment. The red box shows the ATG start site of the AK2 gene. Red letters of amino acid sequence show the difference between wild-type (WT) and the clone. The asterisk indicates stop codon. (**C**) DNA and amino acid mutation sites in AK2 in clone #5 generated from the second trial. Clone #5 was obtained after the second trial of CRISPR/Cas9 treatment. The red box shows the ATG start site of the AK2 gene. Red letters of amino acid sequence show the difference between WT and the clone.

To establish the AK2-deficient model, we used the CRISPR/Cas9 system to delete the AK2 gene in HL-60 cells. The cleavage site was designed to be 43 nucleotides downstream of ATG and the selected guide RNA had a sequence distinct from the AK2 pseudogene [7,8] (Figure 1A). The CRISPR/Cas9 vector was nucleofected into the cells and we obtained four clones. Genomic PCR analysis revealed that these clones had the same 11-base deletion (c. 228_238del11) on one allele, but no mutation on the other allele (Figure 1B). Among them, we selected a clone, #1, which had both a 1-base insertion (c. 221_222insA) and an 11-base deletion (c. 228_238del11) on one allele.

To achieve complete AK2 gene knock-out, clone #1 was nucleofected with the CRISPR/Cas9 vector. Among the resulting clones, AK2 protein expression was determined to be low by Western blot in 18 clones (Figure S1). Genome sequencing of the clones revealed the following: nine of the 18 clones (50%) carried no new mutation; seven (39%) had a new 3-base deletion (c. 232_234delGGC, p. G15del) on one allele and a 1-base insertion on the allele that harbored the 11-base deletion from the first trial; two (11%) had a new 6-base deletion (c. 230_235delAAGGCA, p. K14_G15del) on one allele and the same 1-base insertion (mentioned above) on the other allele (Table 1). These clones did not carry any frameshift mutation. Unfortunately, we could not obtain a complete knock-out clone despite performing CRISPR/Cas9 treatment twice. We decided to use clone #5 and clone #47 for further analyses.

To verify the genomic DNA sequences of clone #5 and clone #47, they were sequenced by next-generation sequencing and their sequences were compared with those of the parental HL-60 cells. Clone #5 showed mutations in exon 1 in both alleles. In addition, one allele displayed a 6-base deletion 37 bases from the start codon. The other allele carried a 1-base insertion 19 bases from the start codon and an 11-base deletion 36 bases from the start codon. Furthermore, intron 4 exhibited a 6-base insertion on one of the alleles. The resultant deduced amino acid sequences at the AK2 N-terminus of clone #5 are shown in Figure 1C. In clone # 47, one allele showed the same sequence as that in the parental HL-60 cells, but the other allele had a 1-base insertion and 11-base deletion, same as in #5. An insertion of 6 bases was found in intron 4, same as in #5 (Table 1).

The deduced AK2 protein sequence encoded by one of the alleles in clone #5 lacks two amino acids near the N-terminus, but the subsequent amino acids show no difference, and the translated protein is supposed to be active. The AK2 protein encoded by the other allele is the result of a frameshift mutation. Since the deduced 17th codon was a stop codon, the AK2 protein was deemed not to be produced (Figure 1B). One of the alleles in clone #47 was deemed to produce the wild-type protein, while no protein should be produced from the other allele due to a frameshift mutation, same as in clone #5. Hence, both the mutants were found to be in a different heterozygous knock-out status. 

### 2.2. Protein Structure Analysis of Clone #5 with the 6-Base Deletion

**Figure 2:**
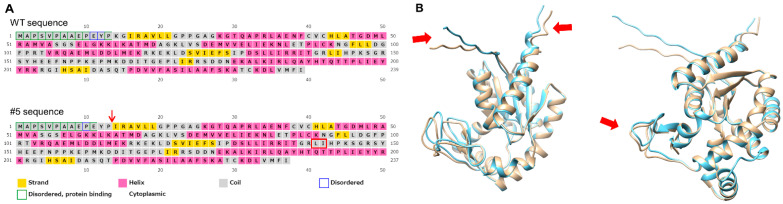
Predicted structures of AK2 protein. (**A**) Secondary structure predictions of AK2 from WT and clone #5. Secondary structures of WT and mutated AK2 were predicted by PSIPRED. Red arrow indicates a deletion site of two amino acids, K and G. Red box indicates a site with changed structure, strand to not strand. (**B**) Tertiary structure predictions and comparison of AK2 from WT and clone #5. Tertiary structure of WT and mutated AK2 were predicted by ColabFold and compared with UCSF Chimera. Blue, WT; yellow, clone #5. Arrows indicate different parts between WT and clone #5.

In clone #5, while one allele of the *AK2* gene was found disrupted, the other allele should produce AK2 protein with a two-amino acid deletion. To evaluate the influence of this deletion on AK2 structure, we compared the predicted AK2 structures of wild-type (WT) and #5 using in silico analysis. First, secondary structures and disordered regions were predicted using PSIPRED and DISOPRED. Disordered regions are those that do not form a fixed three-dimensional (3D) structure and are involved in complex formation, binding to substances, and protein function. The N-terminal disordered domain (blue box) was shorter in #5 than in WT (Figure 2A) and the corresponding protein-binding regions were different between the two. One strand (143L_144I) of the LID domain (142R_178D), which is important for AK2 enzyme activity, was absent in #5. We also predicted and compared the tertiary structures of WT and #5 using ColabFold and UCSF Chimera. The N-terminal disordered domain and the LID domain were found to be different between the two. Overall, these findings suggest that the structural differences in #5 may cause a decrease in AK2 enzyme activity.

### 2.3. Characterization of AK2 Heterozygous Mutants

**Figure 3:**
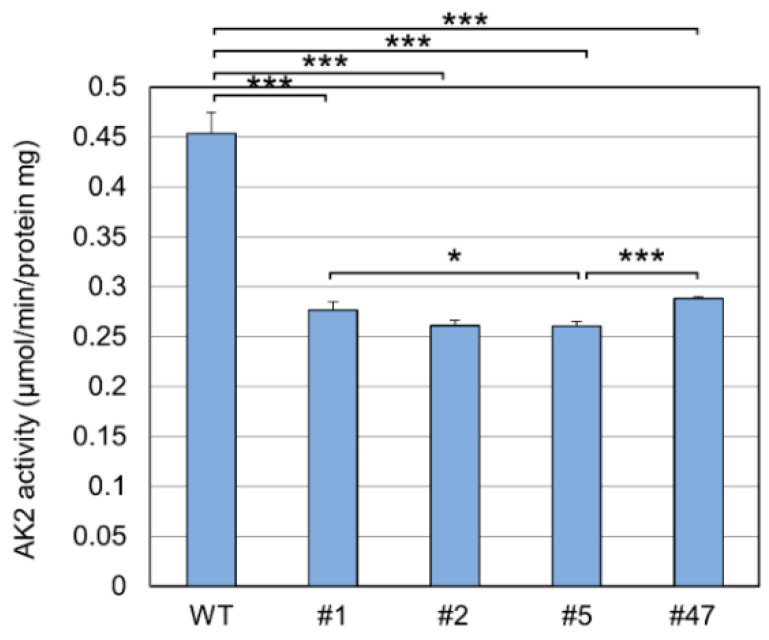
AK2 activity in AK2-deficient clones. AK2 activities of clones were compared with that in WT. Clones #1 and #2 were generated in the first trial. Clones #5 and #47 were generated in the second trial. * *p* < 0.05; *** *p* < 0.001.

To evaluate the influence of mutations on AK2 function, we first analyzed the AK2 enzymatic activities of the two clones, #5 and #47. AK2 activity in the mutants was about 60% of that in the WT (57.5% in #5, 63.8% in #47), and it significantly differed between WT and #5, WT and #47, and #5 and #47 (Figure 3).

On the other hand, the mutants were significantly more viable than the WT (Table 2). Growth rate of each clone was also higher than the WT, while it did not significantly differ between each other. According to these results, AK2 dysfunction might promote cell proliferation and increase cell viability.

Next, we investigated whether the AK2 heterozygous knock-out affected neutrophil differentiation. The two mutants and parental HL-60 cells were cultured for 4 days with 10 µM ATRA and collected on days 0, 1, 2, and 4. The nuclear morphology of the cells was analyzed by Wright–Giemsa staining and used to count the number of mature differentiated cells, segmented neutrophils. At the same time, total RNA was prepared and used for microarray analyses.

**Figure 4:**
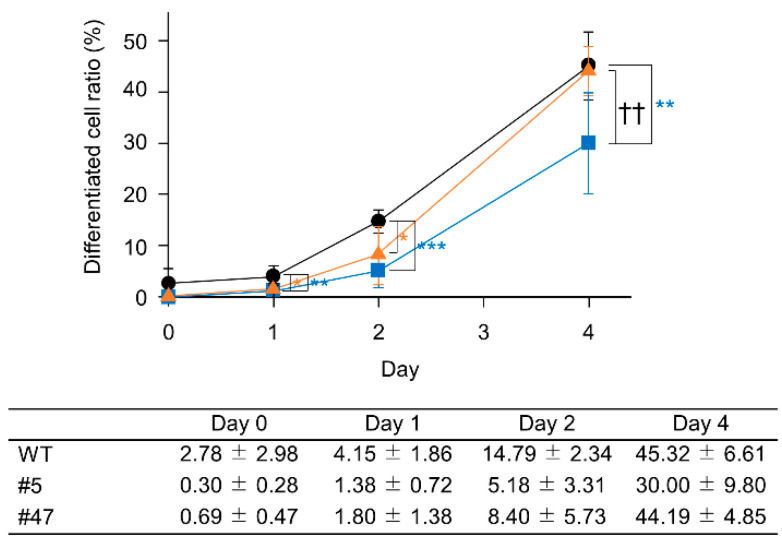
Time course of differentiated cell ratio. The number of differentiated cells relative to the total number of cells following all-trans retinoic acid (ATRA) treatment is shown (HL-60, circle; #5, square; #47, triangle). Each point represents the mean ± standard deviation of three performed experiments. Statistically significant differences between AK2 knock-out cells and HL-60 (* *p* < 0.05; ** *p* < 0.01; *** *p* < 0.001), and between AK2 knock-out #5 and #47 cells (†† *p* < 0.01).

The percentage of segmented neutrophils of the mutants was always lower than that of the WT (Figure 4). Based on the AK2 activity results and estimated differentiation rates, we focused on clone #5 as an AK2 hetero-knockout reticular dysgenesis model system.

**Figure 5:**
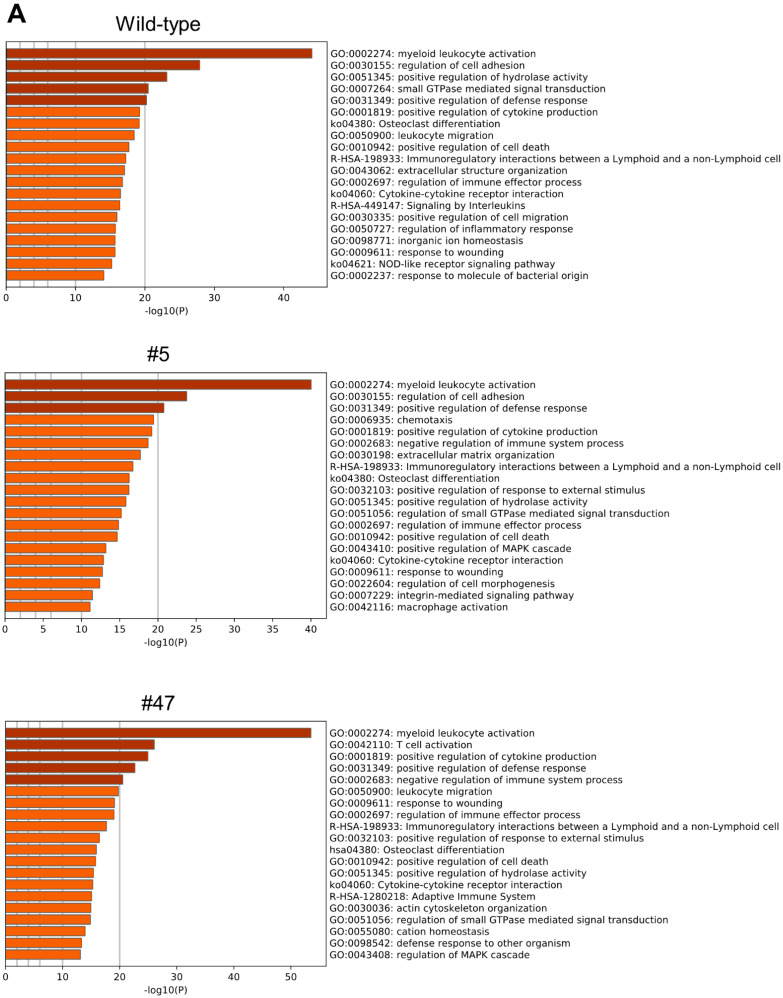

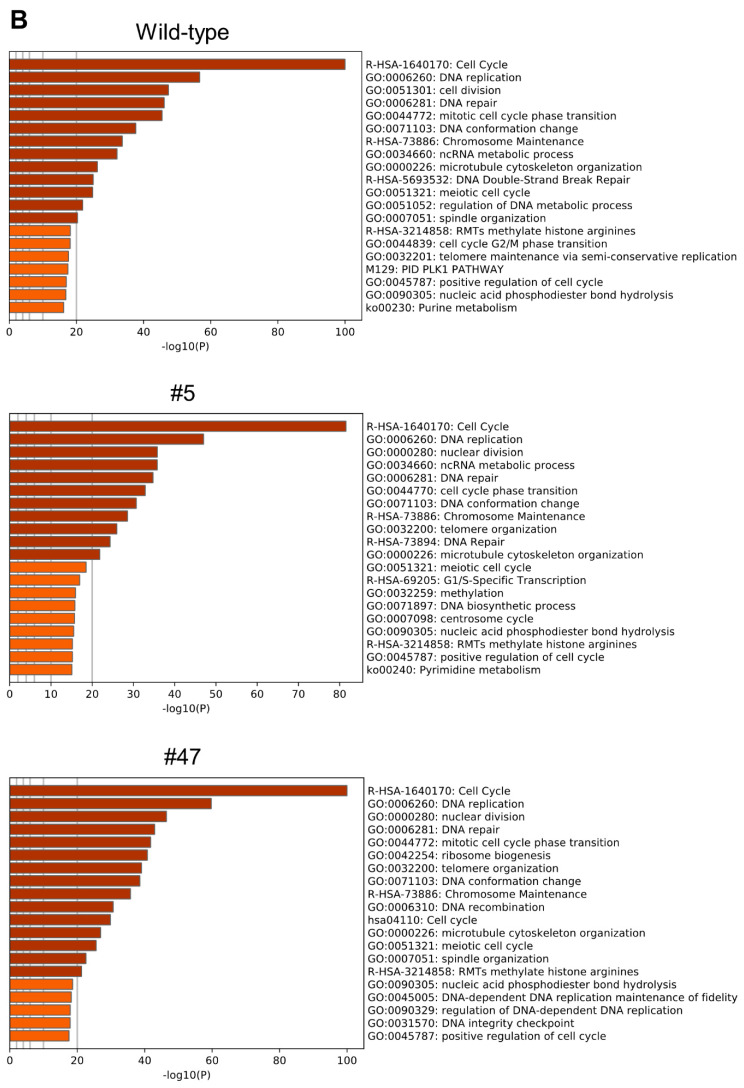
Gene Ontology (GO) analyses of microarray data (**A**) Analysis of the highly expressed genes on day 4 compared with day 0. Twenty gene groups of GO with lowest *p*-values are shown. (**B**) Analysis of the genes whose expression was low on day 4 compared with day 0. Twenty gene groups of GO with the lowest *p*-values are shown.

Microarray analysis was performed to determine the effect of AK2 knock-out on gene expression during differentiation. Genes that were upregulated more than two-fold or downregulated less than half-fold on day 4 compared with day 0 of differentiation were identified using Metascape (Figure 5, Tables S1 and S2).

Among the downregulated genes, clusters such as “cell cycle”, “DNA replication”, “cell differentiation”, and “DNA repair” were detected with higher significance. On the contrary, “myeloid/leucocyte activation”, “cell adhesion regulation”, and “hydrolase activity regulation” comprised the upregulated gene cluster. Similar analyses performed on clone #5 and #47 yielded similar results, suggesting that despite attenuated AK2 activity, ATRA-induced differentiation modulates similar changes in gene expression. However, the levels of expression were lower in the mutants than in the WT. In other words, in the heterozygous knockout of *AK2*, there is no significant difference in the types of genes that respond to ATRA stimulation, but the expression level is reduced, which might delay or weaken the induction of differentiation in the heterozygous AK2 knock-out.

### 2.4. Metabolite Restoration

Metabolic fitness helps a cell to adapt to fluctuating nutrient conditions [9]. The disrupted mitochondrial ATP production in AK2 hetero-knock-out cells might be responsible for the impaired neutrophil differentiation. Therefore, we hypothesized that treating these cells with fructose could complement intracellular ATP by stimulating its production through anaerobic glycolysis, eventually enhancing neutrophil differentiation rates.

**Figure 6:**
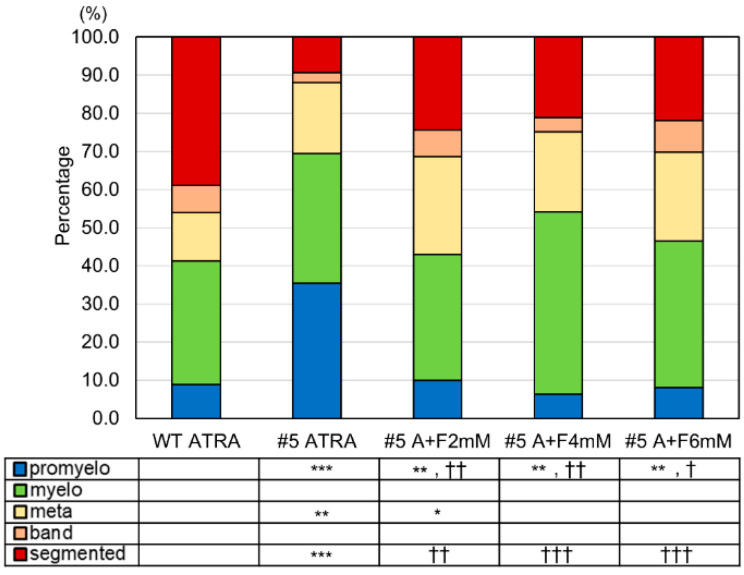
The effects of fructose on neutrophil differentiation in AK2-deficient HL-60 clones. Morphological classification in WT and clone #5 with or without fructose treatment. * *p* < 0.05; ** *p* < 0.01; *** *p* < 0.001 vs. WT ATRA. † *p* < 0.05; †† *p* < 0.01; ††† *p* < 0.001 vs. #5 ATRA.

The segmented neutrophil ratio in the ATRA-treated clone #5 group was significantly lower than in the ATRA-treated WT group (9.3% vs. 38.9%). In contrast, the undifferentiated promyelocyte ratio was significantly higher in clone #5 than in WT cells with ATRA treatment (35.4% vs. 8.9%) (Figure 6). In ATRA-treated clone #5, all three doses of fructose—2 mM, 4 mM, and 6 mM—significantly increased the segmented neutrophil ratios to 24.4%, 21.1%, and 21.9% and significantly decreased the promyelocyte ratios to 10.0%, 6.4%, and 8.1%, respectively. These results suggest that fructose could improve neutrophil differentiation in AK2 hetero-knock-out cells.

**Figure 7:**
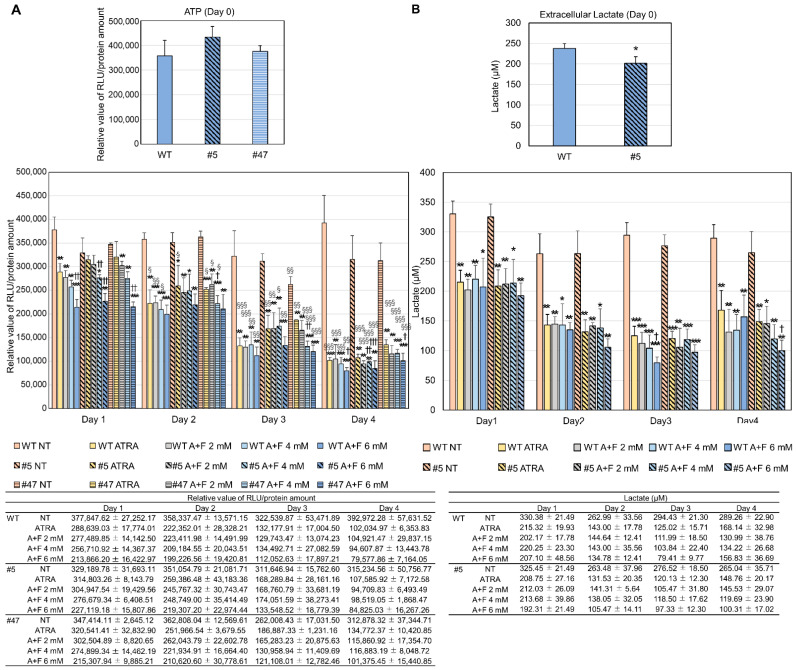
Metabolic changes during neutrophil differentiation with/without fructose in WT and clone(s). (**A**) The ATP levels in clones. (Top) The basic level of cellular ATP in WT and clones. Cells were untreated with ATRA or fructose. No significant difference in these groups. (Bottom) The cellular ATP production with and without fructose treatment during neutrophil differentiation for 4 days. Measured ATP amounts were corrected for each protein content. NT, non-treated; ATRA, all-trans retinoic acid; F, fructose. * *p* < 0.05; ** *p* < 0.01; *** *p* < 0.001 vs. NT. † *p* < 0.05; †† *p* < 0.01; ††† *p* < 0.001 vs. ATRA. § *p* < 0.05; §§ *p* < 0.01; §§§ *p* < 0.001 vs. Day 1. (**B**) The lactate levels in clones. (Top) The basic levels of extracellular lactate in WT and clones. Cells were untreated with ATRA or fructose. * *p* < 0.05. (Bottom) The extracellular lactate levels with and without fructose treatment during neutrophil differentiation for 4 days. * *p* < 0.05; ** *p* < 0.01; *** *p* < 0.001 vs. NT. † *p* < 0.05 vs. ATRA. No significant difference between WT and #5. Each point represents the mean ± standard deviation of three independent experiments.

In addition, we measured the amounts of cellular ATP and extracellular lactate during neutrophil differentiation to confirm the effect of fructose on anaerobic glycolysis and energy production. As in our previous report [2], ATP levels gradually decreased during differentiation in all the experimental groups and those in ATRA-treated groups with/without fructose were significantly lower than in non-treated groups. Furthermore, the ATP levels in groups treated with ATRA plus fructose were tended to be lower than in those treated with ATRA alone, and this trend was dependent on the dose of fructose. Comparing WT and mutants, there are significant differences in ATP levels between WT with ATRA treatment and #47 with ATRA treatment on day 3 and day 4, however, no significant difference otherwise (Figure 7A).

In contrast, lactate level of clone #5 was similar to that of WT (Figure 7B). The extracellular lactate levels were higher in the non-treated group than in the other two. Thus, the levels of intracellular ATP and extracellular lactate during differentiation did not differ between WT and the mutant in most cases.

## 3. Discussion

### 3.1. Characteristics of Heterozygous Mutants

In this study, we established and characterized two AK2-deficient clones. We could only achieve *AK2* heterozygous mutation in HL-60 cells but could not obtain complete *AK2* knock-out cells, possibly because of the following reasons. First, *AK2* knock-out has been reported to be embryonically lethal to mice on day 7 [5,6]. Second, we reported that AK2 is dominantly expressed in day 8 mouse embryos [1], while CKMT1 and CKMT2 are not expressed, and NDPK-D is only expressed at a low level [1]. The same expression pattern can be observed in the human HL-60 cells. Therefore, the complete knock-out of AK2 might be fatal to mouse embryos and HL-60 cells due to a lack of complementation by other energy metabolic enzymes in the mitochondrial intermembrane space.

The bone marrow fluid aspirated from patients with reticular dysgenesis showed a marked increase in promyelocytes [10], whereas HL-60 cells were reported to be arrested in the promyelocyte stage [11]. Therefore, we speculated that the original HL-60 cells might be at a slightly more differentiated stage than promyelocytes, since promyelocytes were present in completely AK2-deficient reticular dysgenesis patients although we were unable to generate complete *AK2*-knock-out HL-60 cells. AK2 is considered critical for the survival of promyelocytes/myelocytes and their differentiation into the next stages. Based on these evidences, we decided to use clone #5 and clone #47 for further study, even though only one allele of the *AK2* gene was disrupted.

### 3.2. Differentiation Activity of AK2 Heterozygous Mutants

Since the HL-60 mutants we generated were heterozygous knock-outs of *AK2*, their enzyme activity is expected to be about half of that in the WT, and our results confirmed this expectation (Figure 3). ATRA-induced neutrophil differentiation of the mutants was delayed or decreased compared with that of the WT (Figure 4). Clone #5 showed that both a neutrophil differentiation rate and AK2 enzyme activity were significantly lower than in WT and clone #47. The slight difference in AK2 activity between the two clones may be the threshold of AK2 activity required for neutrophil differentiation. Microarray analyses revealed that the gene clusters most significantly affected in their expression were similar across mutant HL-60 cells and WT (Figure 5), indicating that the ability to induce differentiation is maintained even if AK2 activity is halved. In a previous study using *Drosophila melanogaster*, we found that AK2 deficiency affected the expression of some components of proteasomal function and the mitochondrial translation machinery [12]. In this study, however, the expression levels of the proteasome subunits were similar between the mutant and the WT cells. The sample used for the *Drosophila* microarray analysis was a homogenate of the whole body of the larvae; thus, the factors contributing to the differences in gene expression may have originated from a mixture of various cell types. Therefore, we could not detect the similarity of gene expression profiles between the *Drosophila melanogaster* model and the HL-60 model used in this study.

Patients with reticular dysgenesis have only a few neutrophils and their AK2 activity is almost lost [3,4]. This study shows that halving the activity of AK2 delays or decreases the induction of neutrophil differentiation (Figure 4 and Figure 6). Neutrophils have a relatively short half-life and a fine balance is maintained in the blood between the number of cells produced and the number of cells dying [13]. The deletion of AK2 perturbs this balance and the rate of degradation overtakes the rate of formation. As a result, the number of neutrophils in the circulating blood may approach zero in patients with reticular dysgenesis.

### 3.3. Possible Roles of Domain Structure

The structural changes caused by amino acid mutations in clone #5—the two-amino acid deletion at the AK2 N-terminus—might affected neutrophil differentiation. As shown in Figure 2B, in silico analysis predicted the AK2 N-terminus to be a disordered domain. In clone #5, the two-amino acid deletion in this disordered domain might impair enzyme activity, protein–protein interactions, and trafficking/localization, resulting in reduced neutrophil differentiation. Furthermore, in silico tertiary structure analysis indicated that the LID domain and the AK2 C-terminus in clone #5 were slightly different from those in WT. The LID domain is required for AK activity to hold the substrate. These structural differences in clone #5 AK2 might explain the attenuated enzymatic activity. However, AK1 and AK5 have a short LID domain and no amino acids corresponding to 143L_144I. Therefore, even if the β-strand is lost in this region, it is unclear how much the structural change would affect enzyme activity. Another possibility is that the two-amino acid deletion affects the G-loop, GPPGAGKGT, in its vicinity. The G-loop is an ATP-binding motif (P-loop) and is critical for AK2 activity [14]. Therefore, it is possible that the deletion of the two amino acids affected the G-loop, resulting in reduced AK2 activity. Further experiments are required to confirm these hypotheses.

### 3.4. Metabolic Restoration by Fructose Supplementation

The effects of fructose vary from cell to cell. For example, fructose increases lipogenesis and is cytotoxic in the liver [15]. However, the effects of fructose on cell differentiation remain unclear. Biochemically, it can be converted into ATP one step earlier by glycolysis in cells other than hepatocytes. The metabolite fructose 1,6-bisphosphate promotes glycolysis by allosteric activation.

We have previously reported that treatment with oligomycin and AK2 small interfering RNA resulted in impaired ATP production, an abnormal unfolded protein response in the endoplasmic reticulum, and impaired neutrophil differentiation due to elevated oxidative stress [1,2]. HL-60 has been reported to express GLUT5, a fructose transporter [16,17]. Therefore, we hypothesized that fructose could increase ATP production by glycolysis and may improve neutrophil differentiation in AK2-deficient HL-60 cells, in which mitochondrial ATP production is impaired.

Morphological analysis revealed that simple ATRA treatment did not promote neutrophil differentiation in clone #5, with its extent being significantly lower than that in WT. Additional fructose treatment increased neutrophil differentiation in this mutant. However, the amount of intracellular ATP and the lactate produced during differentiation did not differ between WT and the mutant. In short, fructose did not increase ATP level by activating glycolysis, but rather restored neutrophil differentiation in a different way.

The pentose phosphate pathway (PPP) is one that branches from glycolysis and is activated by fructose [18]. Ribose 5-phosphate, a product of PPP, is required for DNA synthesis, and PPP has been reported to be required for supplying nicotinamide adenine dinucleotide phosphate and NADPH oxidase function in mature neutrophils [19]. The effect of fructose on cell differentiation has not been clearly reported yet. However, ribose supplementation has been shown to increase neutrophil differentiation. DNA synthesis after differentiation is decreased, but during differentiation, it is highly variable [20]. The previous reports and our results suggest that fructose may promote the PPP, which may induce neutrophil differentiation via unknown mechanisms. This remains to be confirmed in future studies.

## 4. Materials and Methods

### 4.1. Cell Culture and Differentiation

HL-60 human promyelocytic leukemia cells were provided by the RIKEN BioResource Center through the National Bio-Resource Project of the Ministry of Education, Culture, Sports, Science, and Technology, Japan [21,22]. HL-60 cells were maintained in Roswell Park Memorial Institute-1640 (RPMI-1640) medium (05911, Nissui Pharmaceutical Co., Ltd., Tokyo, Japan) with 10% fetal bovine serum (FBS) (Capricorn Scientific, Ebsdorfergrund, Germany, Life Science Production, Bedfordshire, UK). Neutrophil differentiation was induced by treating HL-60 cells (2.5 × 10^5^ cells/mL) with 10 μM ATRA (Sigma, St. Louis, MO, USA) in a flask for 4 days [23].

### 4.2. Preparation of CRISPR/Cas9-Mediated AK2 Heterozygous Mutant Clones of HL-60

The guide RNA to knockout the *AK2* gene was designed using the CRISPR/Cas GeneArt CRISPR Search and Design Tool (Thermo Fisher Scientific, Tokyo, Japan). We selected a 20-nucleotide sequence (5′-CGAGTATCCTAAAGGCATCC-3′) from the candidates, synthesized two oligonucleotides (5′-CGAGTATCCTAAAGGCATCCGTTTT-3′, Reverse 5′-GGATGCCTTTAGGATACTCGCGGTG-3′) (Thermo Fisher Scientific), and inserted them into GeneArt CRISPR Nuclease Vector with OFP Reporter Kit (Thermo Fisher Scientific, A21174), according to the manufacturer’s instructions.

We nucleofected 6 μg or 9 μg of purified CRISPR vector into HL-60 cells, according to our previous report [1]. The next day, single-cell and bulk sorting of orange fluorescent protein-positive cells were performed by JSAN desktop cell sorter (Bay Bioscience, Hyogo, Japan). Bulk culture was grown for a few days and limiting dilution was performed for cloning. Cells were cultured in conditioned medium during cloning.

DNA sequencing was performed using the following primers: Forward 5′-TCGCCGTGCAGTTGGTAAGG -3′, Reverse 5′-TGACCTTGGAGTTCAGCAGG-3′.

To select AK2-edited clones, we purified genomic DNA from each clone and amplified the 5′-end of AK2 coding region by PCR. The PCR products were purified, directly sequenced using a BigDye Terminator v3.1 Cycle Sequencing Kit (Applied Biosystems, Foster, CA, USA), and analyzed with an ABI 3130 Genetic Analyzer (Applied Biosystems).

### 4.3. Western Blot

Western blot analyses were performed as previously described [2]. Briefly, 20 µg of total protein were loaded onto sodium dodecyl sulfate-polyacrylamide gels, electrophoresed, transferred to polyvinylidene difluoride membranes, blocked in 5% skim milk, and probed with the following antibodies: anti-AK2 [24], monoclonal anti-β-actin Clone AC-15 (Sigma), human integrin alpha M/CD11b (238439, R & D systems, Minneapolis, MN, USA) as primary antibodies, and anti-goat HRP IgG (Dako, Glostrup, Denmark), ECL anti-rabbit IgG horseradish peroxidase-linked whole antibody, and anti-mouse IgG (GE Healthcare, Munich, Germany) as second antibodies. Signals were detected using the Immobilon Western Chemiluminescent HRP Substrate (Millipore, Billerica, MA, USA) and Fuji medical X-ray films (Fujifilm, Tokyo, Japan) or ECL Prime Western Blotting Detection Reagent (Cytiva, Tokyo, Japan) and Omega Lum G (Aplegen, Inc., Pleasanton, CA, USA).

### 4.4. Enzymatic Activity

Enzyme assays were performed according to our previous report [2]. Briefly, AK1 and AK2 activities were assayed in the reaction: ATP + AMP ↔ 2 ADP. ADP formation was coupled with pyruvate kinase and lactate dehydrogenase reactions, leading to nicotinamide adenine dinucleotide oxidation. Subsequently, reaction rates of AKs were determined by measuring the decrease in nicotinamide adenine dinucleotide absorbance at 340 nm at 25 ℃. Cells were homogenized and sonicated with lysis buffer (60 mM Tris-HCl pH 7.5, 150 mM NaCl, 5 mM EDTA, 0.2% TritonX-100) on ice. Cell lysates were centrifuged and supernatants were used for the assay. One unit of AKs activity was defined as that required to produce 1 mmol of ADP per minute at 25 ℃. AK2 activity was defined as AKs activity remaining after *N*-ethylmaleimide (AK1 inhibitor) treatment [25], and AK1 activity was determined by subtracting AK2 activity from the total AK activity.

### 4.5. Whole-Genome Analysis (Details of AK2 Locus)

HL-60 and *AK2*-knock-out HL-60 cells were cultured in RPMI-1640 with 10% FBS. HL-60 clone 15 cells were cultured in RPMI-1640, pH 7.8, with 10% FBS. After harvesting the cells, genomic DNA was prepared by the conventional method. Genome analysis was performed by Hiseq X-ten/PE150 (Illumina, San Diego, CA, USA). The analysis results were registered in the Read Archive of the DNA Data Bank of Japan under the accession number DRA013783.

### 4.6. Microarray Analysis during Neutrophil Differentiation

HL-60 cells and *AK2*-knock-out HL-60 cells were treated with 10 µM ATRA and cultured for 4 days. Cells were harvested on days 0, 1, 2, and 4, and total RNA was extracted using QIAGEN’s RNeasy Mini Kit (QIAGEN, Valencia, CA, USA). Gene expression was evaluated using SurePrint G3 Human GE v3 8x60K (Agilent, Santa Clara, CA, USA) at the Advanced Medical Research Institute, Tokushima University. All microarray data were registered with NCBI’s Gene Expression Omnibus under the accession number GSE200827. Gene Ontology analyses were performed using Metascape (https://metascape.org/gp/index.html#/main/step1, accessed on 15 December 2020) [26].

### 4.7. Metabolic Analysis

Cellular ATP content was measured using CellTiter-Glo Luminescent Cell Viability Assay (Promega, Madison, WI, USA), according to the manufacturer’s instructions. Briefly, 100 µL of the cell culture was transferred into a white 96-well plate, incubated at room temperature, and the same amount of CellTiter-Glo reagent was added into the wells. After shaking the plate for 30 s and leaving it to rest for 10 min, the luminescence in the wells was measured using the Cytation 5 microplate reader (BioTek, Santa Clara, CA, USA). Each value was normalized by the total protein amount.

Extracellular lactate was measured using Lactate Assay Kit-WST (DOJINDO LABORATORIES, Kumamoto, Japan), according to the manufacturer’s instructions. A million cells were collected and incubated at 37 ℃ in fresh RPMI-1640 medium containing 1% FBS. Then, 2 µL of medium, 18 µL of distilled water, and 80 µL of working solution were mixed and the secreted lactate was measured at 450 nm using Cytation 5.

### 4.8. Protein Structure Analysis

Secondary structure predictions for WT and mutant proteins were performed using PSIPRED [27], and disordered regions were predicted by DISOPRED [28].

Furthermore, tertiary structures of these proteins were predicted by ColabFold [29]. Based on data obtained by ColabFold, we visualized and superimposed the tertiary structures of WT and mutant proteins using UCSF Chimera [30].

### 4.9. Cytological Staining

Neutrophil-differentiated HL-60 cells were analyzed by staining with Wright–Giemsa solution and May–Grunwald Giemsa solution (Muto Pure Chemicals, Tokyo, Japan), according to the manufacturer’s protocol. The detailed protocol is described in our previous paper [3]. Briefly, cell slides were prepared with Cytospin 4 (Thermo Scientific, Waltham, MA, USA) or smear preparation and stained with Wright–Giemsa or May–Grunwald Giemsa solution.

### 4.10. Statistics 

Each analysis was performed more than three independent materials and experiments conducted under the same experimental conditions. The data was calculated the means ± S.D., respectively. Student’s *t*-test was performed with Microsoft Excel.

## 5. Conclusions

We established a new AK2-deficient model cell line, clone #5, with AK2 hetero-knock-out. Clone #5 showed reduced neutrophil differentiation ability, which is associated with the cryptic formation of a disordered domain. Application of fructose to AK2-deficient cells can be a therapeutic to restore neutrophil differentiation.

## Figures and Tables

**Table 1:** Mutation sites of clones by CRISPR/Cas9.

	Clone	Mutations		cDNA Mutation Sites
		PCR	Whole Genome Analysis	
1st trial	#1	1-base ins and 11-base del/WT	1-base ins and 11-base del/WT	221_222insA and 228_238del11/none
2nd trial	#5	1-base ins and 11-base del/6-base del	1-base ins and 11-base del/6-base del	221_222insA and 228_238del11/230_235delAAGGCA
	#47	1-base ins and 11-base del/3-base del	1-base ins and 11-base del/WT	221_222insA and 228_238del11/none

Del, deletion; ins, insertion.

**Table 2:** Viability of AK2 mutants.

	**Viability (%)**
WT	76.63 ± 2.96
#5	92.57 ± 2.37 **
#47	94.67 ± 1.11 **

Mean ± SD. ** *p* < 0.01 vs. WT.

## Data Availability

The data of whole-genome analysis are deposited in DRA of DDBJ and microarray data are also deposited in GEO of NCBI. Publicly available datasets were analyzed in this study. The data of whole-genome analysis can be found here: [https://www.ddbj.nig.ac.jp/index-e.html] Accession number: DRA013783 (Issue date 4 April 2022), and microarray data can be found here: [https://www.ncbi.nlm.nih.gov/geo/] Accession number: GSE200827 (Issue date 15 April 2022).

## Supplementary Materials

The following supporting information can be downloaded at: https://www.mdpi.com/article/10.3390/ijms232416089/s1.
