# Supplementary material for: The Effect of Heterozygous Mutation of Adenylate Kinase 2 Gene on Neutrophil Differentiation
Source: Int J Mol Sci. 2022 Dec 17;23(24):16089. doi: 10.3390/ijms232416089 (PMC9786915; doi:10.3390/ijms232416089)
Supplement: Supplementary file 1 [file ijms-23-16089-s001.zip › ijms-2042229-supplementary.pdf]

International Journal of
Molecular Sciences

MDPI

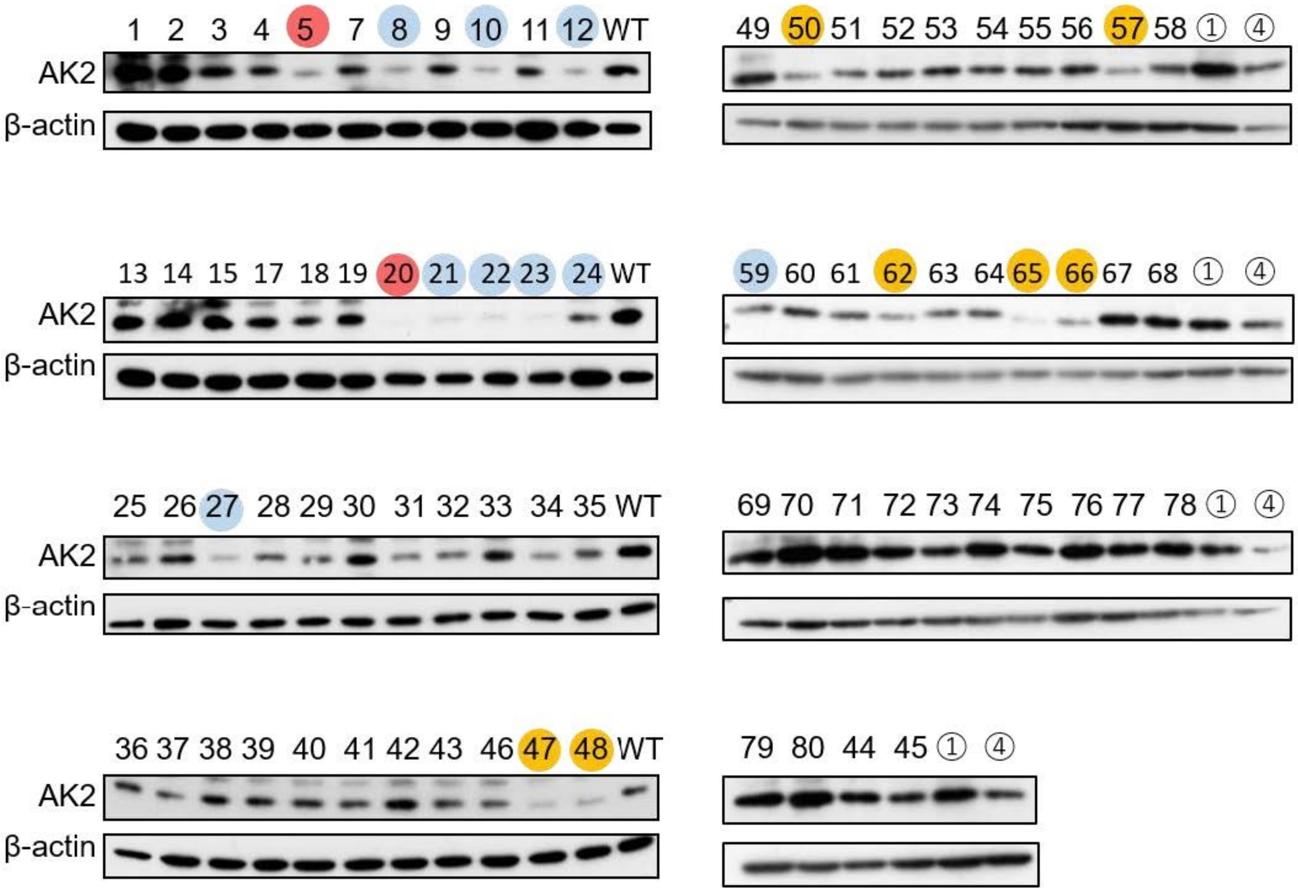

Figure S1: AK2 protein expressions in candidate clones after the second treatment of CRISPR/Cas9

AK2 expression levels in candidate clones with CRISPR/Cas9 treatment twice. β-actin is shown as a loading control. We selected 18 clones and confirmed their genomic DNA sequences by PCR. Blue had no new mutation; orange had a new 3-base deletion on one allele, and an additional 1-base insertion on the allele, wherein the 11-base deletion was induced during the first trial; red had a new 6-base deletion on one allele, and the same 1-base insertion and 11-base deletion on the other allele.

Tables

Table S1: GO analysis of genes that were upregulated on day 4 compared with day 0

GO	Description	Log P
WT		
GO:0002274	myeloid leukocyte activation	-44.05
GO:0030155	regulation of cell adhesion	-27.89
GO:0051345	positive regulation of hydrolase activity	-23.15
GO:0007264	small GTPase mediated signal transduction	-20.47
GO:0031349	positive regulation of defense response	-20.23
GO:0001819	positive regulation of cytokine production	-19.21
ko04380	Osteoclast differentiation	-19.17
GO:0050900	leukocyte migration	-18.45
GO:0010942	positive regulation of cell death	-17.68
R-HSA-198933	Immunoregulatory interactions between a Lymphoid and a non-Lymphoid cell	-17.25
GO:0043062	extracellular structure organization	-17.05
GO:0002697	regulation of immune effector process	-16.75
ko04060	Cytokine-cytokine receptor interaction	-16.48
R-HSA-449147	Signaling by Interleukins	-16.37
GO:0030335	positive regulation of cell migration	-15.96
GO:0050727	regulation of inflammatory response	-15.73
GO:0098771	inorganic ion homeostasis	-15.69
GO:0009611	response to wounding	-15.68
ko04621	NOD-like receptor signaling pathway	-15.21
GO:0002237	response to molecule of bacterial origin	-14.08
#5		
GO:0002274	myeloid leukocyte activation	-40.03
GO:0030155	regulation of cell adhesion	-23.76
GO:0031349	positive regulation of defense response	-20.77
GO:0006935	chemotaxis	-19.41
GO:0001819	positive regulation of cytokine production	-19.18
GO:0002683	negative regulation of immune system process	-18.70
GO:0030198	extracellular matrix organization	-17.70
R-HSA-198933	Immunoregulatory interactions between a Lymphoid and a non-Lymphoid cell	-16.72
ko04380	Osteoclast differentiation	-16.23
GO:0032103	positive regulation of response to external stimulus	-16.20
GO:0051345	positive regulation of hydrolase activity	-15.81
GO:0051056	regulation of small GTPase mediated signal transduction	-15.19
GO:0002697	regulation of immune effector process	-14.84
GO:0010942	positive regulation of cell death	-14.67
GO:0043410	positive regulation of MAPK cascade	-13.19
ko04060	Cytokine-cytokine receptor interaction	-12.86
GO:0009611	response to wounding	-12.74
GO:0022604	regulation of cell morphogenesis	-12.37
GO:0007229	integrin-mediated signaling pathway	-11.43
GO:0042116	macrophage activation	-11.12
#47		
GO:0002274	myeloid leukocyte activation	-53.49
GO:0042110	T cell activation	-26.05
GO:0001819	positive regulation of cytokine production	-24.93
GO:0031349	positive regulation of defense response	-22.65
GO:0002683	negative regulation of immune system process	-20.53

GO:0050900	leukocyte migration	-19.76
GO:0009611	response to wounding	-19.08
GO:0002697	regulation of immune effector process	-18.99
R-HSA-198933	Immunoregulatory interactions between a Lymphoid and a non-Lymphoid cell	-17.68
GO:0032103	positive regulation of response to external stimulus	-16.43
hsa04380	Osteoclast differentiation	-15.91
GO:0010942	positive regulation of cell death	-15.77
GO:0051345	positive regulation of hydrolase activity	-15.38
ko04060	Cytokine-cytokine receptor interaction	-15.28
R-HSA-1280218	Adaptive Immune System	-15.06
GO:0030036	actin cytoskeleton organization	-14.93
GO:0051056	regulation of small GTPase mediated signal transduction	-14.88
GO:0055080	cation homeostasis	-13.92
GO:0098542	defense response to other organism	-13.30
GO:0043408	regulation of MAPK cascade	-13.11

Table S2: GO analysis of genes that were downregulated on day 4 compared with day 0

GO	Description	Log P
WT		
R-HSA-1640170	Cell Cycle	-100.00
GO:0006260	DNA replication	-56.70
GO:0051301	cell division	-47.39
GO:0006281	DNA repair	-46.16
GO:0044772	mitotic cell cycle phase transition	-45.51
GO:0071103	DNA conformation change	-37.70
R-HSA-73886	Chromosome Maintenance	-33.67
GO:0034660	ncRNA metabolic process	-32.11
GO:0000226	microtubule cytoskeleton organization	-26.26
R-HSA-5693532	DNA Double-Strand Break Repair	-25.01
GO:0051321	meiotic cell cycle	-24.83
GO:0051052	regulation of DNA metabolic process	-21.87
GO:0007051	spindle organization	-20.32
R-HSA-3214858	RMTs methylate histone arginines	-18.19
GO:0044839	cell cycle G2/M phase transition	-18.13
GO:0032201	telomere maintenance via semi-conservative replication	-17.61
M129	PID PLK1 PATHWAY	-17.48
GO:0045787	positive regulation of cell cycle	-16.98
GO:0090305	nucleic acid phosphodiester bond hydrolysis	-16.84
hsa00230	Purine metabolism	-15.97
#5		
R-HSA-1640170	Cell Cycle	-81.52
GO:0006260	DNA replication	-46.99
GO:0000280	nuclear division	-35.79
GO:0034660	ncRNA metabolic process	-35.77
GO:0006281	DNA repair	-34.74
GO:0044770	cell cycle phase transition	-32.86
GO:0071103	DNA conformation change	-30.69
R-HSA-73886	Chromosome Maintenance	-28.55
GO:0032200	telomere organization	-25.95
R-HSA-73894	DNA Repair	-24.32
GO:0000226	microtubule cytoskeleton organization	-21.84
GO:0051321	meiotic cell cycle	-18.52
R-HSA-69205	G1/S-Specific Transcription	-16.96
GO:0032259	methylation	-15.95
GO:0071897	DNA biosynthetic process	-15.80
GO:0007098	centrosome cycle	-15.72
GO:0090305	nucleic acid phosphodiester bond hydrolysis	-15.50
R-HSA-3214858	RMTs methylate histone arginines	-15.21
GO:0045787	positive regulation of cell cycle	-15.21
ko00240	Pyrimidine metabolism	-15.09
#47		
R-HSA-1640170	Cell Cycle	-100.00
GO:0006260	DNA replication	-59.74
GO:0000280	nuclear division	-46.44
GO:0006281	DNA repair	-42.97
GO:0044772	mitotic cell cycle phase transition	-41.77
GO:0042254	ribosome biogenesis	-40.78
GO:0032200	telomere organization	-39.07
GO:0071103	DNA conformation change	-38.57

R-HSA-73886	Chromosome Maintenance	-35.78
GO:0006310	DNA recombination	-30.66
hsa04110	Cell cycle	-29.84
GO:0000226	microtubule cytoskeleton organization	-26.90
GO:0051321	meiotic cell cycle	-25.62
GO:0007051	spindle organization	-22.57
R-HSA-3214858	RMTs methylate histone arginines	-21.28
GO:0090305	nucleic acid phosphodiester bond hydrolysis	-18.63
GO:0045005	DNA-dependent DNA replication maintenance of fidelity	-18.22
GO:0090329	regulation of DNA-dependent DNA replication	-17.91
GO:0031570	DNA integrity checkpoint	-17.89
GO:0045787	positive regulation of cell cycle	-17.57
